# 1238. Comparative Activity of Meropenem-Vaborbactam and Ceftazidime-Avibactam Against Multidrug-Resistant *Enterobacter cloacae* from Hospitals in Europe and United States

**DOI:** 10.1093/ofid/ofab466.1430

**Published:** 2021-12-04

**Authors:** Mariana Castanheira, Mariana Castanheira, Jennifer M Streit, Helio S Sader, Dee Shortridge

**Affiliations:** 1 JMI Laboratories, North Liberty, IA; 2 JMI Labortories, North Liberty, Iowa

## Abstract

**Background:**

*Enterobacter* spp. are part of the ESKAPE pathogens that have been recognized as a threat to human health. Among this genus, *E. cloacae* species complex (ECL) is the most common species that causes human infections. ECL can develop resistance to ß-lactams and other antimicrobial classes due to alterations in gene regulatory pathways. We evaluated the activity of meropenem-vaborbactam, ceftazidime-avibactam, and comparator agents against 235 multidrug resistant (MDR) ECL isolates collected in Europe and the US during 2017-2019.

**Methods:**

A total of 2,459 ECL clinical isolates were collected in 40 European and 33 US hospitals. Isolates were susceptibility tested by reference broth microdilution methods and results were interpreted using CLSI, EUCAST, and US FDA breakpoints. MDR was defined as resistant to 3 or more drug classes when applying the CLSI breakpoints.

**Results:**

MDR ECL were observed among 9.6% of the overall isolates. The MDR rate in Europe (12.0%; 155/1,295) was considerably higher than in the US (6.9%; 80/1,164). Meropenem-vaborbactam inhibited 94.5% and 97.4% of the MDR ECL isolates applying CLSI and EUCAST breakpoints, respectively (Table). Meropenem inhibited 77.9%/85.5% of the isolates (CLSI/EUCAST breakpoints). Cefepime inhibited only 26.0%/16.2% of the MDR ECL isolates while piperacillin-tazobactam inhibited only 13.2%/6.4%. Ceftazidime-avibactam inhibited 93.6% of the MDR ECL isolates. Amikacin and tigecycline were the most active non-beta-lactam comparators, inhibiting 91.9% and 80.0% of these isolates using CLSI/US FDA breakpoints. A total of 93.1% of the isolates were intermediate to colistin applying CLSI breakpoints or susceptible using the EUCAST criteria. Meropenem-vaborbactam inhibited 73.5% and 87.8% of the MDR ECL isolates nonsusceptible to meropenem and cefepime, the main therapeutic option against ECL isolates. Ceftazidime-avibactam inhibited 73.5% of these isolates.

**Conclusion:**

In a global surveillance, ECL is the second most common *Enterobacterales* species/species complex displaying MDR and carbapenem-resistance phenotypes, behind only *Klebsiella pneumoniae*. Meropenem-vaborbactam and ceftazidime-avibactam can be important options to treat infections caused by MDR ECL.

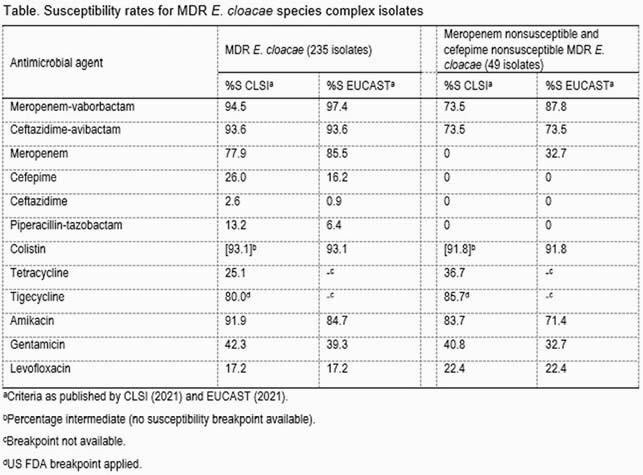

**Disclosures:**

**Mariana Castanheira, PhD**, **AbbVie (formerly Allergan**) (Research Grant or Support)**Bravos Biosciences** (Research Grant or Support)**Cidara Therapeutics, Inc.** (Research Grant or Support)**Cipla Therapeutics** (Research Grant or Support)**Cipla USA Inc.** (Research Grant or Support)**GlaxoSmithKline** (Research Grant or Support)**Melinta Therapeutics, Inc.** (Research Grant or Support)**Melinta Therapeutics, LLC** (Research Grant or Support)**Pfizer, Inc.** (Research Grant or Support)**Qpex Biopharma** (Research Grant or Support)**Shionogi** (Research Grant or Support)**Spero Therapeutics** (Research Grant or Support) **Mariana Castanheira, PhD**, Affinity Biosensors (Individual(s) Involved: Self): Research Grant or Support; Allergan (Individual(s) Involved: Self): Research Grant or Support; Amicrobe, Inc (Individual(s) Involved: Self): Research Grant or Support; Amplyx Pharma (Individual(s) Involved: Self): Research Grant or Support; Artugen Therapeutics USA, Inc. (Individual(s) Involved: Self): Research Grant or Support; Astellas (Individual(s) Involved: Self): Research Grant or Support; Basilea (Individual(s) Involved: Self): Research Grant or Support; Beth Israel Deaconess Medical Center (Individual(s) Involved: Self): Research Grant or Support; BIDMC (Individual(s) Involved: Self): Research Grant or Support; bioMerieux Inc. (Individual(s) Involved: Self): Research Grant or Support; BioVersys Ag (Individual(s) Involved: Self): Research Grant or Support; Bugworks (Individual(s) Involved: Self): Research Grant or Support; Cidara (Individual(s) Involved: Self): Research Grant or Support; Cipla (Individual(s) Involved: Self): Research Grant or Support; Contrafect (Individual(s) Involved: Self): Research Grant or Support; Cormedix (Individual(s) Involved: Self): Research Grant or Support; Crestone, Inc. (Individual(s) Involved: Self): Research Grant or Support; Curza (Individual(s) Involved: Self): Research Grant or Support; CXC7 (Individual(s) Involved: Self): Research Grant or Support; Entasis (Individual(s) Involved: Self): Research Grant or Support; Fedora Pharmaceutical (Individual(s) Involved: Self): Research Grant or Support; Fimbrion Therapeutics (Individual(s) Involved: Self): Research Grant or Support; Fox Chase (Individual(s) Involved: Self): Research Grant or Support; GlaxoSmithKline (Individual(s) Involved: Self): Research Grant or Support; Guardian Therapeutics (Individual(s) Involved: Self): Research Grant or Support; Hardy Diagnostics (Individual(s) Involved: Self): Research Grant or Support; IHMA (Individual(s) Involved: Self): Research Grant or Support; Janssen Research & Development (Individual(s) Involved: Self): Research Grant or Support; Johnson & Johnson (Individual(s) Involved: Self): Research Grant or Support; Kaleido Biosceinces (Individual(s) Involved: Self): Research Grant or Support; KBP Biosciences (Individual(s) Involved: Self): Research Grant or Support; Luminex (Individual(s) Involved: Self): Research Grant or Support; Matrivax (Individual(s) Involved: Self): Research Grant or Support; Mayo Clinic (Individual(s) Involved: Self): Research Grant or Support; Medpace (Individual(s) Involved: Self): Research Grant or Support; Meiji Seika Pharma Co., Ltd. (Individual(s) Involved: Self): Research Grant or Support; Melinta (Individual(s) Involved: Self): Research Grant or Support; Menarini (Individual(s) Involved: Self): Research Grant or Support; Merck (Individual(s) Involved: Self): Research Grant or Support; Meridian Bioscience Inc. (Individual(s) Involved: Self): Research Grant or Support; Micromyx (Individual(s) Involved: Self): Research Grant or Support; MicuRx (Individual(s) Involved: Self): Research Grant or Support; N8 Medical (Individual(s) Involved: Self): Research Grant or Support; Nabriva (Individual(s) Involved: Self): Research Grant or Support; National Institutes of Health (Individual(s) Involved: Self): Research Grant or Support; National University of Singapore (Individual(s) Involved: Self): Research Grant or Support; North Bristol NHS Trust (Individual(s) Involved: Self): Research Grant or Support; Novome Biotechnologies (Individual(s) Involved: Self): Research Grant or Support; Paratek (Individual(s) Involved: Self): Research Grant or Support; Pfizer (Individual(s) Involved: Self): Research Grant or Support; Prokaryotics Inc. (Individual(s) Involved: Self): Research Grant or Support; QPEX Biopharma (Individual(s) Involved: Self): Research Grant or Support; Rhode Island Hospital (Individual(s) Involved: Self): Research Grant or Support; RIHML (Individual(s) Involved: Self): Research Grant or Support; Roche (Individual(s) Involved: Self): Research Grant or Support; Roivant (Individual(s) Involved: Self): Research Grant or Support; Salvat (Individual(s) Involved: Self): Research Grant or Support; Scynexis (Individual(s) Involved: Self): Research Grant or Support; SeLux Diagnostics (Individual(s) Involved: Self): Research Grant or Support; Shionogi (Individual(s) Involved: Self): Research Grant or Support; Specific Diagnostics (Individual(s) Involved: Self): Research Grant or Support; Spero (Individual(s) Involved: Self): Research Grant or Support; SuperTrans Medical LT (Individual(s) Involved: Self): Research Grant or Support; T2 Biosystems (Individual(s) Involved: Self): Research Grant or Support; The University of Queensland (Individual(s) Involved: Self): Research Grant or Support; Thermo Fisher Scientific (Individual(s) Involved: Self): Research Grant or Support; Tufts Medical Center (Individual(s) Involved: Self): Research Grant or Support; Universite de Sherbrooke (Individual(s) Involved: Self): Research Grant or Support; University of Iowa (Individual(s) Involved: Self): Research Grant or Support; University of Iowa Hospitals and Clinics (Individual(s) Involved: Self): Research Grant or Support; University of Wisconsin (Individual(s) Involved: Self): Research Grant or Support; UNT System College of Pharmacy (Individual(s) Involved: Self): Research Grant or Support; URMC (Individual(s) Involved: Self): Research Grant or Support; UT Southwestern (Individual(s) Involved: Self): Research Grant or Support; VenatoRx (Individual(s) Involved: Self): Research Grant or Support; Viosera Therapeutics (Individual(s) Involved: Self): Research Grant or Support; Wayne State University (Individual(s) Involved: Self): Research Grant or Support **Jennifer M. Streit, BS**, **GlaxoSmithKline, LLC** (Research Grant or Support)**Melinta Therapeutics, LLC** (Research Grant or Support)**Shionogi** (Research Grant or Support)**Spero Therapeutics** (Research Grant or Support) **Helio S. Sader, MD, PhD, FIDSA**, **AbbVie (formerly Allergan**) (Research Grant or Support)**Basilea Pharmaceutica International, Ltd.** (Research Grant or Support)**Cipla Therapeutics** (Research Grant or Support)**Cipla USA Inc.** (Research Grant or Support)**Department of Health and Human Services** (Research Grant or Support, Contract no. HHSO100201600002C)**Melinta Therapeutics, LLC** (Research Grant or Support)**Nabriva Therapeutics** (Research Grant or Support)**Pfizer, Inc.** (Research Grant or Support)**Shionogi** (Research Grant or Support)**Spero Therapeutics** (Research Grant or Support) **Dee Shortridge, PhD**, **AbbVie (formerly Allergan**) (Research Grant or Support)**Melinta Therapeutics, Inc.** (Research Grant or Support)**Melinta Therapeutics, LLC** (Research Grant or Support)**Shionogi** (Research Grant or Support)

